# Familial Mediterranean Fever and Hypercoagulability

**DOI:** 10.4084/MJHID.2011.017

**Published:** 2011-05-16

**Authors:** Oshrat E. Tayer-Shifman, Eldad Ben-Chetrit

**Affiliations:** Rheumatology Unit, Division of Medicine, Hadassah-Hebrew University Medical Center. Jerusalem, Israel

## Abstract

Familial Mediterranean fever (FMF) is an autosomal recessive hereditary disease which is characterized by recurrent attacks of fever and peritonitis, pleuritis, arthritis, or erysipelas-like skin disease. As such, FMF is a prototype of autoinflammatory diseases where genetic changes lead to acute inflammatory episodes. Systemic inflammation – in general - may increase procoagulant factors, and decrease natural anticoagulants and fibrinolytic activity. Therefore, it is anticipated to see more thrombotic events among FMF patients compared with healthy subjects. However, reviewing the current available literature and based upon our personal experience, thrombotic events related purely to FMF are very rare. Possible explanation for this discrepancy is that along with the procoagulant activity during FMF acute attacks, anticoagulant and fibrinolytic changes are also taking place. Colchicine which is the treatment of choice in FMF may also play a role in reducing inflammation thereby decreasing hypercoagulability.

## Introduction:

Familial Mediterranean fever (FMF) is an autosomal recessive hereditary disease which is characterized by recurrent attacks of fever and peritonitis, pleuritis, arthritis, or erysipelas-like skin disease.^1^ The gene associated with FMF (MEFV) was isolated in 1997 by two independent groups.^2^,^3^ It encodes a 781 amino acid protein named pyrinmarenostrin, which suppresses inflammation via interaction with peptides and cytokines involved in this process. Mutations in MEFV affect pyrin function, decreasing its suppressor effect of inflammation leading to the eruption of the characteristic acute attack of FMF.^4^ Thus, FMF is a prototype of autoinflammatory diseases where genetic changes lead to acute inflammatory episodes.

Hypercoagulability is a laboratory phenotype whereby *in vivo* activation of clotting, fibrinolysis, endothelial cells and platelets are identified *in vitro* by specialized clotting techniques and by specific antibodies directed at biomarkers of clotting activation and damaged vasculature.^5^ Hypercoagulability may be provoked by inherited or acquired thrombophilia as well as by sepsis, surgery, blood stasis and inflammation.

In general, systemic inflammation may increase procoagulant factors and decrease natural anticoagulants and fibrinolytic activity.^6^ Furthermore, many cytokines involved in inflammation are also known to trigger coagulation.^7^ As a prototype inflammatory disease in FMF, levels of acute phase reactants and cytokines, such as CRP, IL-1, IL-6 and IL-8, are significantly elevated during and between attacks.^8^ Therefore, one expects to see more thrombotic events among these patients compared with healthy subjects. However, reviewing the current available literature and based on our personal experience in our clinic, the impression is that events of thrombosis related purely to FMF are very rare. Case reports about thrombotic events in FMF are almost always on patients with renal disease, amyloidosis and proteinuria or other concomitant thrombophilias.^9^–^11^

We review the available literature about coagulability in FMF and try to understand why thrombotic events are rare in this disease despite its being a typical inflammatory disease.

## Fibrinogen:

Fibrinogen contributes to thrombosis risk in different ways and is important in regulating inflammatory response.^12^ Significant differences in levels of fibrinogen among FMF patients during an attack, patients free of attack and controls were found in one study, where the highest levels were found in patients during attacks, medium levels in patients free of attack and the lowest levels in healthy controls.^13^ In another study, no statistically significant difference in serum fibrinogen was found in attack-free periods of FMF patients compared with healthy controls, but the levels were remarkably higher in patients during the acute attacks.^14^ Thus, in FMF - especially during acute attacks - levels of fibrinogen can be high. Since this protein is an important precursor for fibrin, which is essential for the final clotting pathway, this situation may theoretically enhance coagulation.

## Prothrombin Time and Thrombin Time:

Thrombin, formed by activation of the coagulation cascade, is essential to promote hemostasis but also stimulates several cell functions, including chemotaxis and mitogenesis. Thrombin not only converts fibrinogen to fibrin, it also acts as a proinflammatory agent, resulting in a positive feedback loop of the inflammocoagulatory response.^15^ Thrombin time (TT) as well as prothrombin time (PT) were shortened in attack-free periods in FMF patients compared with controls, suggesting an increased baseline inflammatory condition with an increased state of hypercoagulabilty.^14^ Contradictory results were reported in another study where the PT in FMF patients during an attack period was significantly prolonged compared with patients during their attack-free periods or with a healthy control group. This observation was accompanied by increased levels of the acute phase reactants suggesting discordance between these two processes - thrombosis and inflammation. However, it should be emphasized that in the later study, PT levels varied largely among the patients of all groups, especially in the attack group.^13^ These contradictory results should be resolved in additional studies. In the meantime, the role of thrombin as a procoagulant factor in FMF should await further investigation.

## D-dimer:

D-dimer is formed as a result of the plasmin-mediated, proteolytic degradation of cross-linked fibrin.^16^ In general, D-dimer levels are increased during any clinical condition where both clot formation and subsequent fibrinolysis take place.^17^ Elevated D-dimer levels were found in FMF patients during the acute attacks. Between the attacks, D-dimer levels were still elevated compared with healthy controls but far less than those in patients with acute attack.^13^ However, in another study, D-dimer levels between controls and attack-free patients were found to be similar, suggesting that these components are elevated mainly during acute FMF attacks.^14^ Thus, increased D-dimer may indicate a fibrinolytic process taking place during the acute attack or an expression of increased acute phase reactants due to the inflammatory episode.

## Plasminogen Activator Inhibitor 1:

Plasminogen activator inhibitor 1 (PAI-1) is produced by endothelial cells and inhibits fibrinolysis. PAI-1 is also considered to be one of the markers of endothelial dysfunction. An increase in PAI-1 causes hypofibrinolysis and results in a hypercoagulable state.^18^ In a single study which measured levels of PAI-1 among FMF patients, significant low levels were found during the attack-free period compared with the acute attack period.^13^ This observation suggests that during the acute event elevated PAI-1 may increase coagulability. However, the exact role of this peptide in predisposing thrombosis in FMF should await further studies.

## Tissue Plasminogen Activator:

Tissue plasminogen activator (tPA) is the trigger of the fibrinolytic activity, and fibrinolytic pathways must be activated along with the coagulation activation.^19^ Significantly higher levels of tPA were found in FMF patients during attacks than in controls, and statistically insignificant higher levels were found in attack-free periods in FMF patients compared with controls.^13^ This observation may indicate that during inflammation there is a persistent fibrinolytic activity which counterbalances coagulation during the ongoing inflammation of FMF attacks.^6^ Thus, tPA may serve as a counter regulator of the previous components which contribute to the procoagulant status.

## P-selectin:

P-selectin is a marker of thrombocyte activation. Serum levels of P-selectin did not differ significantly between FMF patients during the attack or attack-free periods. However, these levels were significantly lower in both patient groups compared with controls. Again, the low levels of P-selectin might reflect a response mechanism to counteract the hypercoagulable state in FMF patients. Nevertheless, since the blood sampling was late (in the FMF attacks), one should be cautious in drawing firm conclusions.^13^

## Protein C Activity:

Activated protein C (APC) is a natural anticoagulant that plays an important role in coagulation homeostasis by inactivating the procoagulation factors Va and VIIIa. In addition to its anticoagulation functions, APC also has cytoprotective effects such as anti-inflammatory, anti-apoptotic, and endothelial barrier protection.^20^

Protein C activity decreased in FMF patients during attack-free periods compared with healthy controls. This finding may indicate the problem in suppressing inflammatory response through the protein C pathway in the acute attack.^14^ Furthermore, reduction of APC may increase hypercoagulability in FMF patients.

## Fibronectin and Thrombospondin:

Fibronectin (FN) and thrombospondin (TSP) are extracellular matrix proteins. Fibronectin, apart from being an adhesive solid phase element, has many different roles in wound healing and cellular adhesion. More recently, FN has emerged as a player in platelet thrombus formation and diseases associated with thrombosis, including vascular remodeling, atherosclerosis and cardiac repair following myocardial infarction.^21^

Thrombospondin is a glycoprotein that interacts with a wide variety of molecules, including heparin, fibrin, fibrinogen, platelet cell membrane receptors, collagen, and fibronectin. It also plays a role in platelet aggregation, tumor metastasis, vascular smooth muscle growth and tissue repair in skeletal muscle following crush injury. As such, it is another extracellular matrix product which is primarily found in the granules of thrombocytes. However, it can also be synthesized and secreted by many inflammatory cells, including neutrophils. Both fibronectin and thrombospodin may play a role in promoting hypercoagulability in addition to their role in inflammation. Plasma FN and TSP levels during acute FMF attacks were significantly higher than after resolution of the acute attacks. In addition, significant correlations were observed between both FN and TSP levels and CRP and WBC counts in two different studies.^13^,^22^ On one hand, the above findings may suggest that FN and TSP are factors potentially able to enhance hypercoagulability during the acute attacks. On the other hand, their high correlation with acute phase reactants may reflect their important roles in the inflammatory process during the attacks rather than their procoagulant effect on this condition.

## vW Factor and Factor VIII:

Von Willebrand factor (vWF) is a large multimeric glycoprotein present in blood plasma and produced constitutively in endothelium, megakaryocytes and subendothelial connective tissue.^23^ Its primary function is binding to other proteins, in particular Factor VIII, and it is important in platelet adhesion to wound sites. Endothelial damage due to inflammation triggers the coagulation cascade. In a study where levels of factors VIII and vWF were measured during FMF attacks and during free-attack periods no difference was found. Furthermore, these results did not differ from those found in healthy controls.^13^ It seems that these two factors do not play any role either in inflammation or in coagulation in FMF.

## Thrombocytes and MPV:

Although thrombocytes can be used as a marker for acute phase reactants, and MPV levels may be the first to increase in response to inflammation, no differences in thrombocyte count and MPV were found between FMF patients and controls.^13^

## Other Factors:

Increased levels of prothrombin fragments F1+2 were found in attack-free periods in FMF patients compared with healthy controls. No differences were found in levels of activated protein C resistance (APCR), antithrombin or anti cardiolipin antibodies in FMF patients and controls.^14^

## Discussion:

Many studies have shown that in FMF inflammation exists not only during the acute attacks, but also between attacks. Increased CRP and serum amyloid A (SAA) and elevated cytokines levels between attacks reflect an ongoing sub-clinical inflammation during these periods.^24^ The coagulation system is closely related to the inflammatory system and it is known that systemic inflammation and elevated cytokines are capable of triggering coagulation.

The above-mentioned studies which dealt with coagulability changes in FMF patients shed some light on the association between coagulation and inflammation during the acute attacks and in between them. Most of these investigations show that in FMF - especially during the acute attacks - some parameters of coagulation are abnormal. This theoretically may increase the risk for thrombotic events in FMF patients. Nevertheless, reviewing the English literature for thrombotic events in these patients disclosed very few case reports. A single case report presented a child with FMF who experienced a stroke. An extensive thrombophilia work-up revealed that the patient had concomitant multiple inherited and acquired risk factors for thrombosis.^9^ In other cases of thrombotic events in FMF patients, concomitant renal disease or amyloidosis was also found.^10^ It seems that the presence of proteinuria in these cases served as a second hypercoagulable hit, due to deficiency of antithrombin causing development of thrombosis. A recent study reported two FMF patients who developed Budd Chiari syndrome. Thorough investigation revealed that one of the patients was homozygous for MTHFR mutation (677C>T), whereas the other carried a single mutation in the factor V Leiden (FVL).^11^ The authors suggested that “FMF should be regarded as a possible additional thrombotic risk factor in such cases”.

Following the identification and isolation of the MEFV gene, several studies searched for a possible role of MEFV mutations in hypercoagulability. In fact a study by Rabinovitch et al. found that in patients with Behcet’s disease who carried the MEFV mutation M694V, there were more vascular and thrombotic events compared with BD patients who did not carry this mutation.^25^ Since Behcet’s disease is characterized by thrombotic events due to endothelitis and vasculitis, the question is raised as to the exact role or contribution of FMF and MEFV mutations in these patients who are *a priori* prone to develop thrombosis.

The above cases show that FMF alone is not a direct cause for thrombosis and, in fact, in all the above cases there was an additional procoagulant background which led to the thrombotic complication.

This observation raises the question regarding the explanation for the scarce reports on thrombotic events in patients with FMF only.

One possible answer could be that along with the ongoing inflammation in FMF leading to the procoagulant activity during the acute attacks and in between them, anticoagulant and fibrinolytic changes are also taking place. Thus, there may be a balance between coagulation and fibrinolysis *in vivo*. The higher than normal tPA levels during attack-free periods in FMF patients may indicate the importance of such persistence of the fibrinolytic activity for the balance of ongoing inflammation and coagulation and may be the key for the prevention of future thrombosis.^6^ The changes in P-selectin may play a similar anticoagulant role.^13^

An additional possible explanation is that all the above factors which are associated with hypercoagulability in fact also serve as inflammatory components. It may well be that during the acute attack of FMF they are consumed or used for the purpose of inflammation so that nothing is left for their role in the coagulation pathway. It seems that during an acute FMF attack the inflammatory process overcomes the coagulation process.

Another question which might be raised is whether colchicine – which is taken continuously by FMF patients - has any role in the coagulation pathway.

The observation that the highest levels of D-dimer were found during attacks in FMF patients who did not use colchicine suggests that colchicine decreases fibrinolytic activity by suppressing inflammation. Colchicine treatment is known to reduce the frequency and intensity of attacks in FMF, and it apparently also lowers the level of circulating fibrin, thereby reducing coagulation and later fibrinolysis.^13^ Colchicine treatment also lowers levels of soluable E- and L-selectin in FMF and Behcet’s patients (26, 27) and was shown to diminish the qualitative expression of E-selectin on endothelium, and the quantitative expression of of L-selectin on neutrophils.^28^

**In summary,** the studies reviewed herein have just started revealing the procoagulant activity in FMF and its relationship to the acute attacks. Although it seems that coagulability is increased and enhanced during these attacks and in between them, the patients do not experience increased thrombotic events.

Yet the current studies are few and in some cases the results are conflicting. Larger studies are needed to confirm the observations about the changes in coagulation in FMF patients. Furthermore, large case age matched control prospective studies are require to disclose the potential association of coagulation parameters with MEFV mutations.
